# *In Vitro* Evaluation of Spider Silk Meshes as a Potential Biomaterial for Bladder Reconstruction

**DOI:** 10.1371/journal.pone.0145240

**Published:** 2015-12-21

**Authors:** Anne Steins, Pieter Dik, Wally H. Müller, Stephin J. Vervoort, Kerstin Reimers, Jörn W. Kuhbier, Peter M. Vogt, Aart A. van Apeldoorn, Paul J. Coffer, Koen Schepers

**Affiliations:** 1 University Medical Center Utrecht, Wilhelmina Children’s Hospital, Division of Pediatrics, Utrecht, The Netherlands; 2 Utrecht University, Department of Chemistry, Utrecht, The Netherlands; 3 University Medical Center Utrecht, Department of Cell Biology, Utrecht, The Netherlands; 4 Medical School Hannover, Department of Plastic, Hand and Reconstructive Surgery, Hannover, Germany; 5 University of Twente, MIRA Institute for Biomedical Technology and Technical Medicine, Department of Developmental Bioengineering, Enschede, The Netherlands; Texas A&M University Baylor College of Dentistry, UNITED STATES

## Abstract

Reconstruction of the bladder by means of both natural and synthetic materials remains a challenge due to severe adverse effects such as mechanical failure. Here we investigate the application of spider major ampullate gland-derived dragline silk from the *Nephila edulis* spider, a natural biomaterial with outstanding mechanical properties and a slow degradation rate, as a potential scaffold for bladder reconstruction by studying the cellular response of primary bladder cells to this biomaterial. We demonstrate that spider silk without any additional biological coating supports adhesion and growth of primary human urothelial cells (HUCs), which are multipotent bladder cells able to differentiate into the various epithelial layers of the bladder. HUCs cultured on spider silk did not show significant changes in the expression of various epithelial-to-mesenchymal transition and fibrosis associated genes, and demonstrated only slight reduction in the expression of adhesion and cellular differentiation genes. Furthermore, flow cytometric analysis showed that most of the silk-exposed HUCs maintain an undifferentiated immunophenotype. These results demonstrate that spider silk from the *Nephila edulis* spider supports adhesion, survival and growth of HUCs without significantly altering their cellular properties making this type of material a suitable candidate for being tested in pre-clinical models for bladder reconstruction.

## Introduction

Various diseases of the genitourinary system, such as bladder exstrophy and neurogenic bladder, can cause damage to the bladder and often require reconstructive surgery to prevent urinary retention, incontinence or renal damage. To prevent upper tract damage and achieve social continence, augmentation cystoplasty is performed in which the bladder is reconstructed by enlargement of the bladder with a piece of autologous intestine [1,2]. However, serious adverse events are associated with the use of intestine for augmentation cystoplasty such as metabolic disorders, mucus production, bladder rupture and malignancy [3,4].

To avoid the use of intestine in the urinary tract and prevent associated complications, researchers developed a number of strategies to reconstruct the bladder using both natural (e.g. collagen), synthetic (e.g. polyglycolic acid (PGA)) or a combination of both materials (e.g. decellularized submucosa such as small intestine submucosa (SIS) or composite collagen-PGA) as a scaffold. However, these materials are subject to structural, mechanical, functional and biocompatibility failure [5–12]. A highly suitable and promising biomaterial for bladder reconstruction is silk, which has a slow degradation rate as well as high tensile strength and elasticity [13–17]. A very widely studied silk is cocoon silk from the silkworm *Bombyx mori* (*B*. *mori*). Seth et al. already demonstrated the *in vivo* use of *B*. *Mori* silk-based biomaterials for augmentation cystoplasty by means of salt-leaching methods [18]. Although this silk can easily be produced on a large scale, sericin coating surrounding the fibers induces T cell mediated allergic reactions as well as macrophage activation. Furthermore, degumming of the silk to remove the sericin coating makes the silk very susceptible to tensile deformation [15,19–21].

In contrast to *B*. *Mori* silk, spider silks generally lack a sericin coating and offers great improvements in tensile strength, toughness and extension [15,17,22–24]. Spider major ampullate gland-derived (MA) dragline silk of *Nephila species*, being one of the most well studied spider silks, provides this combination of outstanding strength, elasticity, biocompatibility, biodegradability and high temperature resistance [14,25]. In fact *Nephila species*-derived spider silk has better tensile strength, is less stiff and has a higher % strain at break than degummed *B*.*Mori* silk [15]. As spider silk consists of mostly nonpolar amino acids alanine (27%), glycine (20%) and Prolin (13%) as well as the amino acids glutamate, arginine, serine, aspartic acid, isoleucine, lysine, phenylalanine, threonine, tyrosine and valine (1–9%), it is highly biocompatible [26–28]. This makes spider silk a good candidate for reconstruction of a complex organ such as the bladder, which endures high elongation and tensile forces.

*In vitro* studies show great potential for native spider silk in the reconstruction of various organs [22,29,30]. *In vivo* studies testing the biocompatibility of spider silk in animal models show a mild to low inflammatory reaction and a degradation rate of approximately six months [22,24,31–33]. Although current drawback of native spider silk for *in vivo* use is the difficulty of large-scale production, ongoing advances in recombinant spider silk are likely to solve these issues [34].

The promising results using spider silk as a biomaterial in preclinical animal models, encouraged us to investigate native *Nephila edulis* spider-derived MA dragline silk, as a potential biomaterial for bladder reconstruction. For this purpose, we here studied the cell–material interactions of primary human urothelial cells (HUCs), which are multipotent cells able to form the mucosa of the bladder with spider silk [35–38]. We describe the successful culturing of HUCs on native dragline silk matrices and provide thorough assessment of the effect of spider silk on survival, expansion and differentiation of HUCs *in vitro*, identifying it as a promising biomaterial for being test for *in vivo* bladder reconstruction.

## Materials and Methods

### Breeding and handling of spiders and frame preparation

*Nephila edulis* spiders were bred at the Medical School Hannover, Dept. of Plastic, Hand and Reconstructive Surgery. Spider MA dragline silk was collected from female spiders by a harmless fixation technique on a foam platform as previously described [27]. Stainless steel weaving frames were bent into square frames with a side length of 0.5–0.8 cm and spider MA dragline silk was woven onto the frames at a fixed rate (i.e. 30 revolutions per minute) for 7.5 minutes on each side thus creating a cross weaved mesh. Frames were autoclaved at 120°C for 1 hour and placed separately in 24-wells plates in sterile PBS at 37°C, 5% CO_2_, for several days prior to use to remove the 10–20 nm outermost lipid coat of the fibers [39]. For coating experiments, spider silk frames were incubated in 1 ml fibronectin (Sigma; 1:100 in PBS (10 μg/ml)) or 1 ml Poly-L-Lysine (ScienCell; 1:667 in PBS) at 37°C, 5% CO_2_ for 2 hours and washed twice with PBS prior to use.

### Indirect determination of the mechanical forces of the spider silk fibers

Deformation by forces occurring while the silk is reeled on the weaving frames was determined by measuring the distances of the opposing wires of the frame before and after silking with n = 12. Forces necessary to restore the original size were measured with a spring balance, indicating indirectly the mechanical forces of the spider silk fibers.

### Cell culture

HUCs were cultured as described by the cell provider (ScienCell) in serum-free Urothelial Cell Medium (UCM) (ScienCell). For culturing HUCs on spider silk, passage 5–6 HUCS (1x10^4^/frame) were incubated for 1.5 hours at 37°C, 5% CO_2_ in 20 μl UCM to promote cell attachment. Subsequently, 1 ml of UCM was added to each well and media were changed every three days. T24 cells were cultured in IMDM medium (Gibco) supplemented with 1% penstrep (Gibco) and 10% FBS (PAA Cell culture Company) [40]. RT112 cells were cultured in fully supplemented RPMI+ Glutamax medium (Gibco) [41].

### Cytocompatibility assay

To determine cytocompatibility, HUCs were cultured in unconditioned UCM, 1 mM H_2_O_2_ (VWR International), or conditioned medium generated by immersing a spider silk frame or a piece of SIS (Biodesign Cook Medical) in UCM for 2 days at 37°C, 5% CO_2_. Cytotoxicity was measured with the Vibrant® MTT cell proliferation assay (Molecular Probes) using a Microplate Reader (model 3550, Bio-Rad).

### Histology

Frames were fixed in 4% formaldehyde (FA) (Merck) for 24 hours at RT and spider silk meshes were embedded in paraffin. The 4 μm thick paraffin sections were stained with Haematoxylin (Klinipath) and Eosin (Merck) and imaged with a Nikon DXM1200 digital camera.

### (Immuno)fluorescence staining

For visualization of filamentous actin and nuclei, samples were fixed in 4% FA at RT, permeabilised with 5% Saponin and 1% BSA and stained with Phalloidin and DAPI (all from Sigma) and imaged using confocal microscopy (LSM700, Zeiss). To determine cell expansion, five fixed fields of vision (FOV, enlargement 10x) images were made with an EVOS® cell imaging system (Life Technologies) per sample and the amount of DAPI positive nuclei per FOV were counted using ImageJ software. To determine viability, a live/dead cell imaging assay (Molecular Probes) was performed. To determine HUC growth in time we measured the metabolic activity of HUCs by resorufin fluorescence using Presto Blue (PB, Molecular Probes) and a SpectraMax (Molecular Devices).

### Scanning electron microscopy

For visualization of adhesion of HUCs on spider silk frames, constructs were fixed in 4% FA for 24 hours at RT and dehydrated as described earlier [42]. Ethanol was replaced with anhydrous acetone (acetone and 1% acidified 2.2-dimethoxypropane (DMP), 1:100) [43]. The samples were dried using a CPD-030 Leica critical point drying apparatus (Leica Microsystems, Vienna, Austria) and coated with 6 nm Pt/Pd using a sputter coater 208HR (Cressington Scientific Instruments Ltd., Chalk Hill Watford, England, UK). Specimens were viewed in an XL30 scanning electron microscopy (SEM) equipped with a field emission gun (FEI Europe, Eindhoven, The Netherlands).

### RNA isolation and qRT-PCR

After six days of culturing, total RNA from HUCs, T24, and RT112 was isolated using the RNeasy mini kit (Qiagen, Hilden, Germany) and cDNA was synthesized using the iScriptTM cDNA Synthesis kit (Bio-Rad) in a T3000 Thermocycler (Biometra). QPCR was performed using SYBR green Supermix (Bio-Rad) using the indicated primers (Table 1, Sigma) and the MyiQ Single Color RT PCR detection system (BioRad). Relative gene expression was calculated using the 2^-ΔΔCt^ method normalized to β2M levels for each individual sample.

**Table 1:** Primer sequences.

Gene	Forward primer	Reverse primer
Vimentin	ACCAACGACAAAGCCCGCGT	CAGAGACGCATTGTCAACATCCTGT
E-cadherin	CACCACGTACAAGGGTCAGGTGC	CAGCCTCCCACGCTGGGGTAT
N-Cadherin	AGTCACCGTGGTCAAACCAATCGA	TGCAGTTGACTGAGGCGGGTG
Collagen 1	CCCCAGCCACAAAGAGTCTAC	TGATTGGTGGGATGTCTTCGT
Cytokeratin 18	CATGCAAAGCCTGAACGACC	ATTTGCGAAGATCTGAGCCCT
Cytokeratin 19	TGAGGAGGAAATCAGTACGCTG	TTGGCTTCGCATGTCACTCA
Cytokeratin 17	GGAGCAGCAGAACCAGGAATA	CTTGTACTGAGTCAGGTGGGC
Cytokeratin 7	TCTTTGAGGCCCAGATTGCTG	GCAGCATCCACATCCTTCTTC
Integrin α2	TTAGCGCTCAGTCAAGGCAT	CGGTTCTCAGGAAAGCCACT
Integrin β1	CCGCGCGGAAAAGATGAATTT	CCACAATTTGGCCCTGCTTG
Integrin α6	CAAATGCAGGCACTCAGGTTC	AGCCTTGTGATATGTGGCATCA
Integrin α5	AAGACTTTCTTGCAGCGGGA	GCCACCTGACGCTCTTTTTG
CD44	AGCAACTGAGACAGCAACCA	AGACGTACCAGCCATTTGTGT

### Flow cytometry

After six days of culturing, cells were collected and stained using anti-CD90, anti-CD49f (both BD Pharmingen), anti-CD44 (Immunotools) antibodies and SytoxBlue (Invitrogen) to exclude dead cells. Cells were measured on a FACSCanto II flow cytometer (BD Biosciences). Data analysis was performed using FlowJo version 7.6 (FlowJo LLC).

### Statistical analysis

The mean and standard deviation (SD) were calculated with Microsoft Excel 2007 (Microsoft Corporation, Schiphol, The Netherlands) and Graphpad Prism 5® (GraphPad Software Inc., LaJolla, California, USA). Statistical analysis was performed using the unpaired student’s t-test and differences with p≤0.05 were considered statistically significant.

## Results

### Mechanics of spider silk weaving frames

To determine roughly the strain forces of the spider silk fibers on the weaving frame caused by the reeling process, deformation forces of the whole weaving frames were measured and calculated for single fibers. The length of one silk fiber used for one side of the frame was approximately 3 meters and the mean distance between single meshes (n = 297) was 32.42 ± 22.27 μm and the 95% confidence interval was 29.88–34.95 μm. Deformation of the steel frames by pulling forces occurred with a mean of 6.69% +/- 2.08% (SD) of the original side length but could be considered as tolerable, as they did not exceed 10%. Force needed to reverse deformation (F_measured_) were also ruled out by measuring the force necessary to reverse deformation and deformation strength (S_calculated_) was calculated by dividing F_measured_ by thickness d of straight wire with equation (1): *S*_*calculated*_ = *F*_*measured*_ *d* ^−1^ [Pa]. As mean, F_measured_ was measured to 1.55 N +/- 0.66 N (SD) and S_calculated_ with d = 0.7 mm was calculated to 2.21 MPa.

### Spider silk supports HUC adhesion and survival

**Fig 1:**
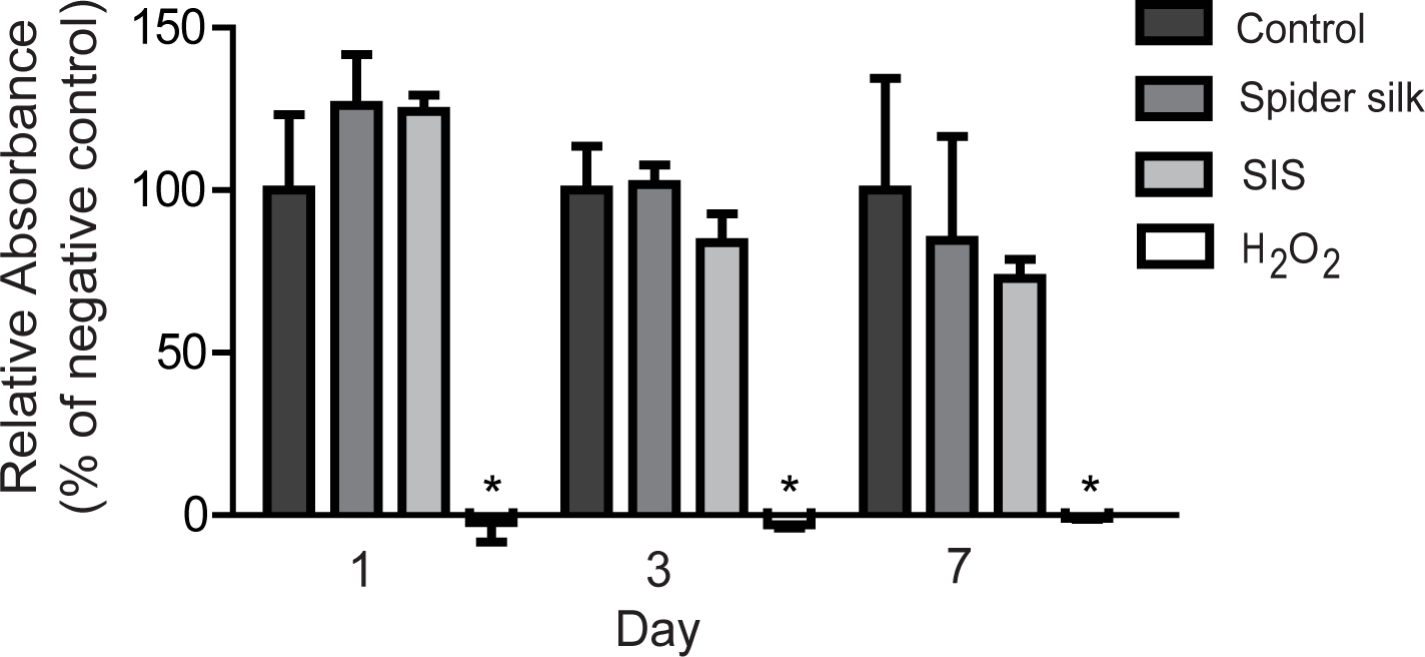
Spider silk extract is not cytotoxic for HUCs. MTT assays showing HUC viability and expansion when exposed to spider silk and SIS conditioned medium, UCM (negative control) and H_2_O_2_ at indicated days of culturing. Relative MTT absorbance as compared to negative control values per time point are shown for each sample. Data are means ±SD (n = 3). * p≤0.05

To test whether spider silk can be used as a potential biomaterial for bladder reconstruction, we exposed HUCs to an extract of spider silk, or SIS, which is the current gold standard in bladder reconstruction, and measured cytotoxicity by MTT assay. Conditioned medium of spider silk or SIS did not significantly affect HUC expansion (Fig 1), indicating that spider silk extract has no cytotoxic effects on HUCs.

**Fig 2:**
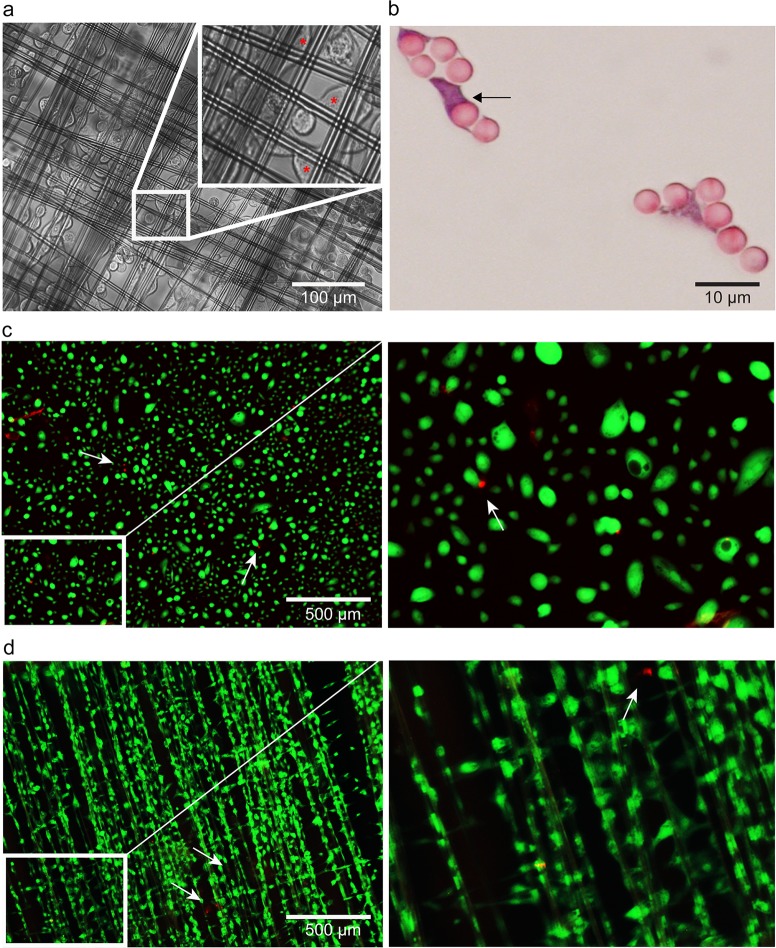
HUC adhesion and survival on spider silk. (a) Representative phase contrast microscopy images of HUC-seeded spider silk matrices at 90 minutes post seeding. Inserts show non-adhered cells with a rounded appearance, red asterisks indicate HUCs adhered to spider silk. (b) Representative H&E staining of paraffin cross-section of HUC-seeded spider silk matrices at 7 days post seeding. HUCs appear purple and spider silk fibers pink. Arrows indicate the HUCs’ ‘bridging’ capacity. (c-d) Representative microscopy images of live/dead staining of HUCs cultured on glass coverslips (c) or spider silk (d) in which live and dead cells stain with green cytoplasmic fluorescence and red nucleic fluorescence (white arrows), respectively. Inserts show magnification of the indicated (white box) area.

To determine whether HUCs are able to adhere to spider silk, we used light microscopy. Within 90 minutes after seeding, HUCs adhered to the spider silk matrices and cell bodies stretched longitudinally alongside the spider silk fibers and bridged the gaps between the spider silk fibers (Fig 2A). H&E staining of paraffin cross-sections confirmed this ‘bridging’ capacity (Fig 2C and 2D, black arrows). Live/dead assay performed on HUCs seeded on either spider silk or glass at 6 days post seeding revealed that dead (red) cells were only detected occasionally (white arrows, Fig 2E and 2F). Together, these results demonstrate that spider silk matrices allow adherence and survival of HUCs.

### HUC adhesion induces actin-rearrangements and filopodia development

**Fig 3:**
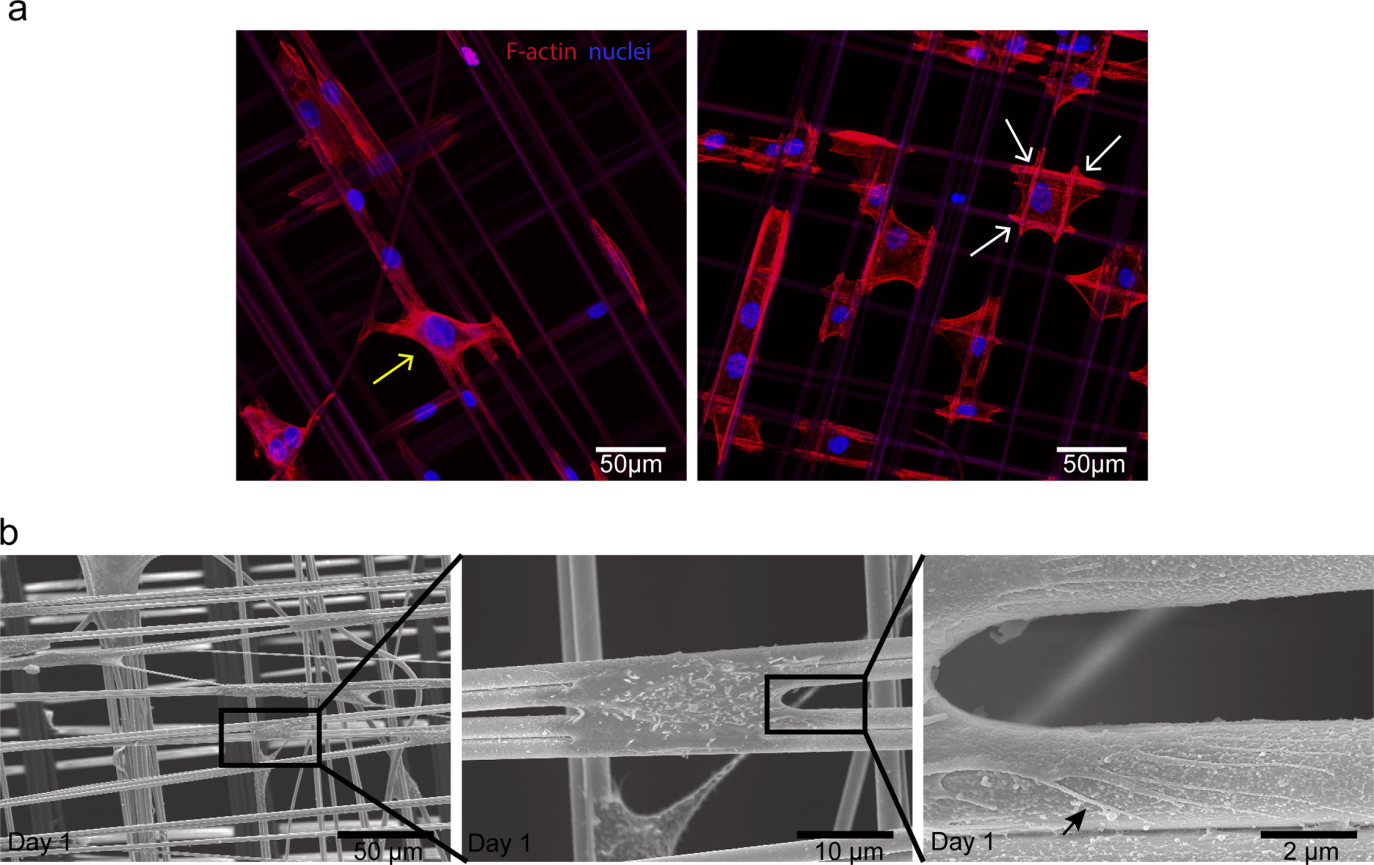
HUC adhesion is associated with actin-rearrangements and filopodia development. (a) Representative confocal images of DAPI (blue) and phalloidin (red) stained HUC-seeded spider silk matrices at day 1 post seeding. White arrows indicate some of the attachment sites of HUCs concentrated around the fiber mesh that stain brightly with fluorescing phalloidin, the yellow arrow indicate cells capable of bridging the gap between two spider silk fibers. (b) Representative electron micrographs of spider silk matrices at day 1 post HUC seeding. Consecutive magnifications are shown in sequence. The black arrow indicates one of the many filopodia present on the fiber surface protruding from a single HUC.

To obtain insight in the processes involved in HUC adherence, we stained HUCs seeded on spider silk matrices with DAPI and phalloidin to detect the nucleus and filamentous actin, respectively. Confocal imaging confirmed that HUCs stretched in various directions (Fig 3A). Furthermore, actin filaments specifically accumulated in proximity to the spider silk fibers as indicated by the bright intracellular phalloidin staining in these areas (Fig 3A, white arrows), suggesting an important role for actin-rearrangements in adhesion of HUCs to spider silk. Subsequent SEM revealed that HUCs wrap numerous lengthy filopodia around the spider silk fibers (Fig 3B), which were determined to be approximately 2 μm thick. Together, these results demonstrate that HUCs rearrange their cytoskeleton and form filopodia while adhering to the spider silk fibers.

### Spider silk allows HUC expansion

**Fig 4:**
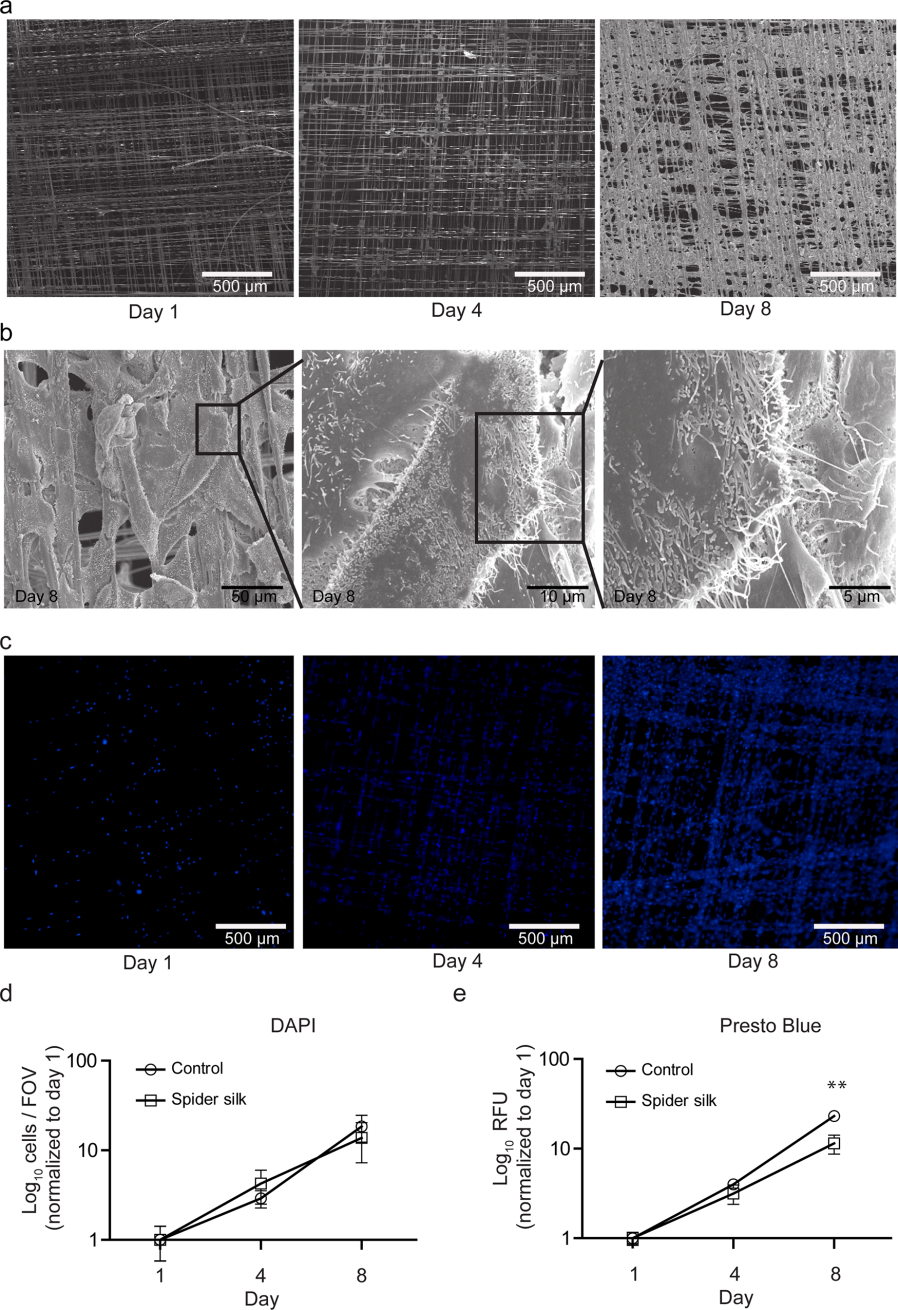
Spider silk supports expansion of HUCs. Visualization of HUC growth on spider silk frames by SEM (a-b) or DAPI immunostaining (c-d). Representative images of HUC growth at the indicated days post seeding (a and c). Detailed SEM images at day 8 post seeding (b). Quantification of HUC expansion by counting DAPI positive cells (d) or by Presto Blue fluorescence in relative fluorescence units (RFU) (e). Data are means ±SD (n = 3). ** p≤0.01 between spider silk and control.

To investigate HUC growth over time we performed SEM analysis at different time points after seeding (Fig 4A). Within 8 days HUCs progressively covered most of the spider silk fibers (Fig 4A). High magnification electron micrographs at 8 days post seeding showed multiple layers of cells covering the spider silk matrix (Fig 4B).

Quantification of cell nuclei per field of view (FOV) in immunofluorescence images of DAPI-stained HUCs (Fig 4C and 4D) revealed no difference in cell growth between HUCs grown on spider silk or plastic. To confirm this, we followed HUC growth by measuring the metabolic activity of HUCs by resorufin fluorescence. Initially (day 1–4) no significant differences were observed in HUC growth on either plastic or spider silk (Fig 4E). However, at day 8 HUC growth on spider silk was slightly decreased. Together, these results show that spider silk allows the expansion of HUCs into a multilayered HUC network within 8 days of culture.

### Coating spider silk improves HUC adhesion but does not compromise growth

**Fig 5:**
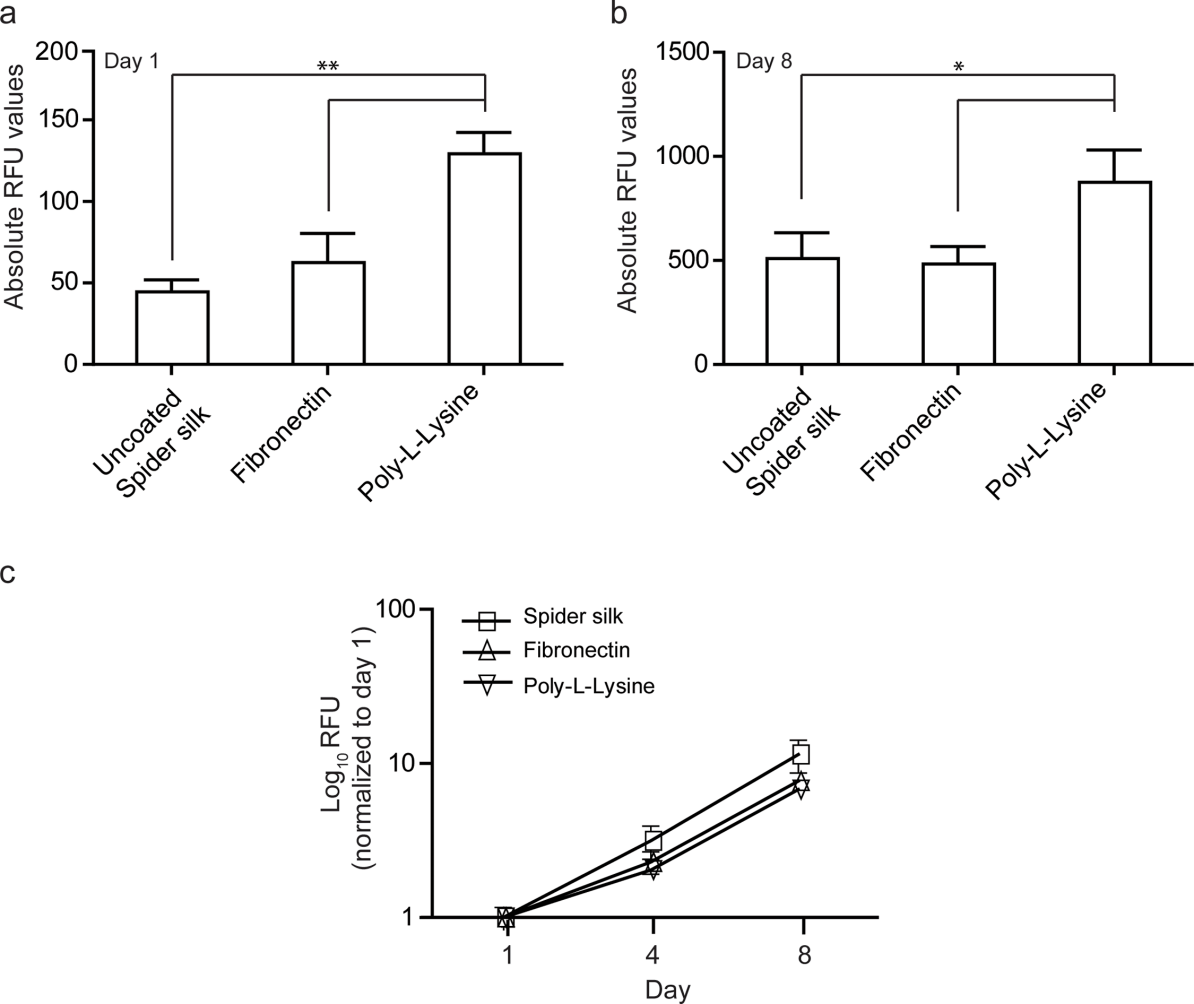
Coated spider silk enhances cell adherence but diminishes growth. Presto Blue-based quantification of the amount of viable HUCs on spider silk coated with the indicated substrates. (a) RFU at day 1 post seeding as percentage of HUCs cultured on uncoated spider silk. (b) Absolute RFU values at day 8. (c) RFU between day 1 to 8 normalized to day 1 demonstrating cell growth. Data are means ±SD (n = 3). * p≤0.05, ** p≤0.01.

To determine whether HUC adherence and growth can be further improved we applied bioactive coatings on the spider silk. One day post-seeding, spider silk coated with poly-l-lysine showed a significant increase in the number of viable HUCs as compared to fibronectin and non-coated spider silk (+/- 3 fold), indicating that poly-L-lysine can improve the adherence of HUCs to spider silk (Fig 5A). Subsequent analysis over an 8 days period revealed that HUCs adhering to either coated or uncoated spider silk did not show any significant difference in expansion (Fig 5B). When normalized to day 1, relative cell growth was highest in HUCs cultured on non-coated spider silk compared to fibronectin and poly-l-lysine coated spider silk (Fig 5C).

### Spider silk-exposed HUCs show minimal gene expression changes

**Fig 6:**
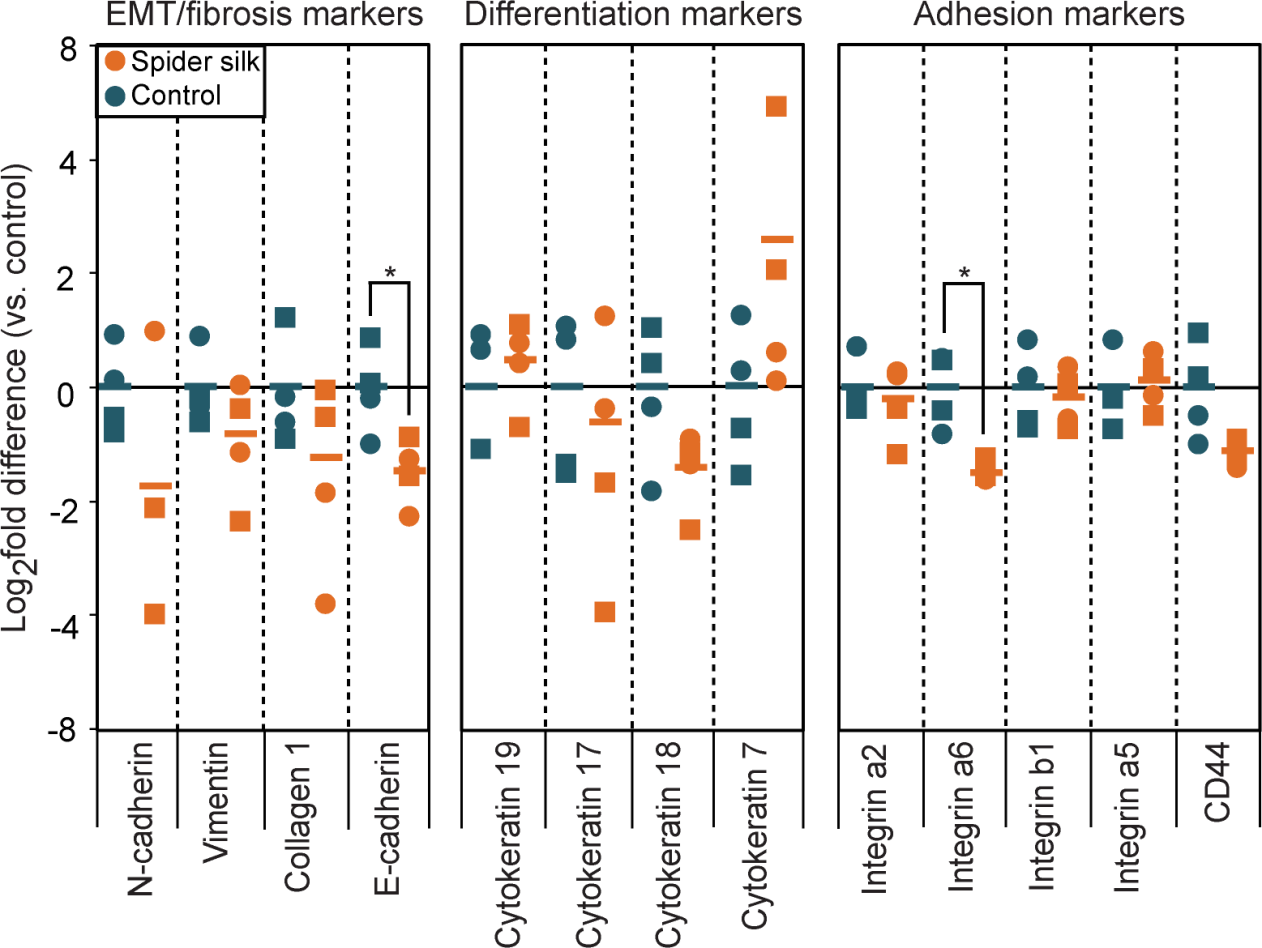
Spider-silk exposure minimally affects the gene expression profile of HUCs. qRT PCR-based gene expression analysis of EMT/fibrosis markers, differentiation markers and adhesion markers in HUCs cultured on plastic or spider silk for six days. Data are normalized to β2M and expressed as log_2_ fold change relative to average level in HUCs cultured on plastic. Data are means ±SD (n = 4). * p≤0.05, ** p≤0.01.

To determine whether exposure to spider silk leads to molecular alterations in HUCs, we analyzed the mRNA expression levels of various genes involved in epithelial-to-mesenchymal transition (EMT), fibrosis, differentiation and adhesion by qRT PCR. Spider silk-exposed HUCs only showed small decreases (1.5–1.7 fold) in the levels of E-cadherin and Integrin α6 (*ITG6*), which are involved in cell-cell or cell-matrix interactions, but showed no differences in any other genes involved in EMT, fibrosis or differentiation (Fig 6).

Taken together, these results indicate that spider silk has marginal effects on the studied gene expression profile of HUCs.

### Spider silk-exposure maintains the undifferentiated nature of HUCs

**Fig 7:**
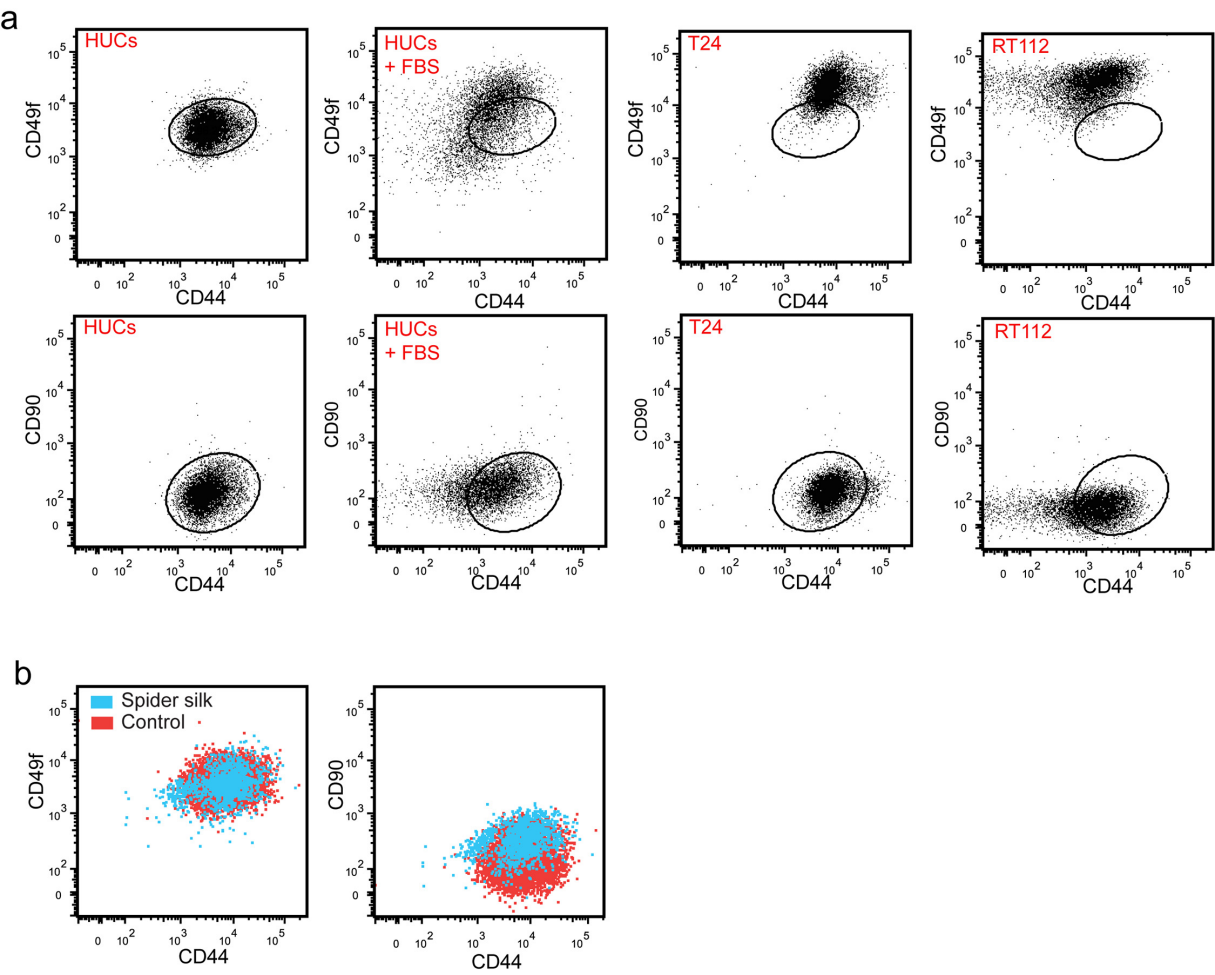
Slight differentiation of a subpopulation of spider silk-exposed HUCs. (a) Representative FACS plots of T24 cells, RT112 cells, HUCs and HUCs treated with fetal bovine serum (FBS) and overlays of HUCs cultured on plastic or spider silk (b) that were stained for CD90, CD44, CD49f and Sytox Blue. Dead cells and doublets were excluded.

We used a recently reported flow cytometry-based approach to determine the effects of spider silk on HUC differentiation 39 [44]. This method uses surface markers to identify urothelial stem cells as CD90^+^CD44^+^CD49f^+^ cells, basal cells as CD90^-^CD44^+^CD49f^+^, intermediate cells as CD90^-^CD44^-^CD49f^+^ and umbrella cells as CD90^-^CD44^-^CD49f^-^. In line with their reported basal cell phenotype HUCs were positive for both CD44 and CD49f and negative for CD90 (Fig 7A) [45]. Furthermore, FBS-induced differentiation resulted in a decrease in CD44 and CD49f expression [46,47]. In accordance with Ho *et al*., who implicated basal subtype cancers with poor clinical outcomes and better outcomes in intermediate subtype cancers, the high-grade metastatic human T24 showed a higher expression level of CD44 than the non-metastatic well differentiated RT122, respectively [44].

Spider silk exposure induced a significant (p≤0.05, n = 3) increase in the CD90 expression HUCs. Furthermore, in line with the trend towards a decrease in CD44 mRNA expression (Fig 6B), HUCs cultured on spider silk contain a small subpopulation of CD44 negative cells (Fig 7), suggesting that spider silk induces the differentiation of a small fraction of silk-exposed cells. Together, these results demonstrate the usefulness of the flow cytometry-based approach to identify various bladder cell subsets and indicate that HUCs for most part maintain their basal phenotype upon exposure to spider silk.

## Discussion

Previous studies have shown that various scaffold materials can be used to reconstruct the bladder. Although SIS or composite materials (such as collagen-PGA) showed promising results, long term side effects including reduced bladder capacity, fibrosis and graft shrinkage has shown that these biomaterials are not optimal for bladder augmentation [9–12]. Given the reported outstanding strength, elasticity, biocompatibility and biodegradability of spider MA silk, which exceed characteristics of B. Mori silk, the scope of this study was to test the cell—material interactions of HUCS with spider MA dragline silk from *Nephila edulis* and provide an in-depth assessment on cell behavior of HUCs when in contact with spider silk with respect to adherence, survival, cell growth and differentiation.

### Mechanical consideration

To achieve a scaffold usable for tissue engineering purposes, dental straight wire was bent to square weaving frames. Straight wire is a medical product which can be sterilized and shows neither *in vivo* nor *in vitro* cytotoxicity. With a bending strength of 1.9 x 10^4^ MPa, a Young’s modulus of 1.7 x 10^3^ MPa and a bending stiffness of 670 kPa (according to manufacturers’ manual), straight wire holds certain stability to theoretically withstand greater bending forces. However, a distinct deformation could be observed, which occurred obviously due to the forces caused by the silking process. As this deformation was reversible, i.e. an elastic deformation for which Hooke’s Law holds, it can be stated that forces that occur during silking spiders, in general surpass bending stiffness of 670 kPa, but below Young’s modulus of 1.7 x 10^3^ MPa. Interestingly, S_calculated_ was calculated 2.21 MPa, further substantiating this thesis. It has to be considered that these values are very roughly as with the experimental setting in this pioneering study measurement errors were relatively high. Nevertheless, even if tolerances of >100% were considered, S_calculated_ was still in the expected range. Interestingly, our results correspond very well with values of forces occurring during rearing process (i.e. spinning) described in literature [26,48]. While the most exact measurement probably was performed by Pérez-Rigeiro et al., the measured spinning force was constant in the mentioned studies around 5.5–7.5 mN [48,49]. It has to be considered that F_measured_ needed to restore original frame had to be divided by 240 fibers to achieve F_measured_ for one single fiber (F_single fiber_), resulting in a F_single fiber_ of 6.46 mN +/- 2.75 mN (SD), matching exactly to the spinning forces determined by Pérez-Rigeiro et al. Additionally, relative deformation was measured and calculated to 6.68%, indicating that no greater deformation rates occurred even with high tolerance rates. This deformation also influenced neither mesh pattern nor cell growth, thus we could found no disadvantages by the minor deformations that occurred.

### Cytocompatibility, adhesion and cell expansion

We report that spider silk extract is non-toxic for HUCs [22,23,27,29,30]. Furthermore, HUCs that adhere to spider silk showed good viability comparable to HUCs cultured on glass. In all our experiments the spider silk matrices were pre-incubated in PBS to remove the 10–20 nm outermost lipid coat of the fibers thereby revealing the glycoprotein layer of spider silk containing fine fibrils and promoting cell adherence [39]. In contrast to what was previously reported that fetal calf serum (FCS) is necessary for HUCs to adhere to SIS [50], HUCs can be cultured on spider silk without addition of FCS. H&E and phalloidin staining revealed that the adhering HUCs stretch along the spider silk where filamentous actin was specifically localized to areas where HUCs were in close contact to spider silk. The HUCs bridge the gaps between the individual fibers in the spider silk mesh, indicating an attractive morphology of the spider silk for HUCs to adhere to and good geometry of mesh fabrication (i.e. inter-fiber distance). Furthermore, SEM revealed that HUCs adhered to the spider silk by using filopodia. Recent studies demonstrate that integrin-extracellular matrix contacts occur in fibroblasts at the tips of filopodia which organize into focal adhesions and serve as anchor points for the cytoskeleton [51]. Together this could indicate that in HUCs a similar process occurs in which integrin and actin containing filopodia form the initial cell-cell and cell-extracellular matrix contacts, and could be responsible for cell attachment and growth on spider silk.

Our study revealed that HUCs grown on spider silk show a slightly decreased expansion as compared to HUCs grown on tissue culture polystyrene, which is in accordance with studies demonstrating lower growth rates of fibroblasts when cultured on spider silk matrices as compared to cover slips [27]. This decreased expansion of cells on spider silk might be related to the requirement of cells to bridge the gaps in between the spider silk fibers when grown on silk, whereas on plastic the surface area to attach and expand is much larger. This is also supported by SEM images demonstrating HUCs growing near confluent on spider silk after 8 days of cultivation. In contrast, previous studies testing the growth of HUCs on SIS demonstrate generally show much slower growth [52–54]. Furthermore, the reported expansion of HUCs on spider silk in this study is much faster than the reported growth of HUCs on uncoated B. Mori silk constructs [55]. Together, this indicates that spider silk is a biomaterial that properly supports the growth of HUCs, which might even be superior over other biomaterials.

Similar to what has been reported by Franck *et al*. for silkworm-derived silk, coating spider silk with fibronectin, which is expressed in and near the urothelial basement area, did not improve HUC adherence and slowed down the growth of HUC cells [55]. The only method that improved adherence of HUCs to the spider silk was coating the spider silk with Poly-L-Lysine, a synthetic compound which is highly positively charged and which is also often used for culturing HUCs on tissue culture plastic and is well known to improve adhesion of cells [56]. However, since spider silk without any additional bioactive coating spider silk already showed good biocompatibility, adherence and growth support for primary HUCs, we focused on analyzing HUC behavior on uncoated spider silk.

### Differentiation

Although it is known that substrate properties such as surface chemistry and matrix stiffness can influence cellular behavior and differentiation [57], qRT PCR analysis revealed that many genes are expressed at similar levels in HUCs cultured on spider silk or on tissue culture plastic. The only genes that showed significant differential expression between HUCs cultured on either plastic or spider silk were the genes encoding E-cadherin and integrin α6, which are both involved in cell-cell and cell-extracellular matrix interactions. Downregulation of ITGa6 was only marginal and only observed at the mRNA level. In contrast, CD49f protein expression, which is the product of the ITGa6 gene and was analyzed by flow cytometry, remained unchanged upon exposure to spider silk. Although it has been described that E-cadherin loss can be an indication of EMT, we did not observe changes in the mesenchymal genes encoding Vimentin and N-cadherin. Overall, these results suggest that exposure of HUCs to spider silk only minimally affects the behavior of HUCs.

We used a recently described flow cytometry-based method that uses CD44 as well as the cell surface markers CD49f and CD90 to identify different cell types within the normal urothelium to further elucidate the effect of spider silk on HUC behavior and differentiation [58]. As expected, HUCs showed a basal cell phenotype (i.e. CD90^-^CD44^+^CD49f^+^) [45]. In line with the trend towards decreased CD44 mRNA expression in spider silk exposed HUCs, flow cytometric analysis revealed that exposure of HUCs to silk results in decreased levels of CD44 protein albeit in only a small subpopulation of HUCs, rather than in the total cell population. So rather than adjusting to a specific differentiated state, this indicates that most silk-exposed HUCs remain undifferentiated and keep their basal phenotype. This is beneficial for clinical application, since the *in vivo* microenvironment should allow the proper differentiation of HUCs to epithelial bladder cell layers that mimics the native bladder wall, similar to previous studies in which undifferentiated HUCs were seeded onto collagen-based scaffolds [10].

## Conclusions

We have shown that native spider MA dragline silk from *Nephila edulis* supports the adhesion, survival and growth of HUCs, while maintaining their undifferentiated state. In combination with the reported outstanding strength, elasticity, biocompatibility and biodegradability of spider MA silk [13–17], these results establish spider MA dragline silk from *Nephila edulis* as a promising biomaterial for bladder reconstruction. In order to make a functional construct capable of miction and urine storage, it is of great importance to incorporate a layer of smooth muscle cells (SMCs) into the construct. Atala et al. demonstrate successful engraftment of an a-cellular scaffold seeded with both HUCs and SMCs in patients. In this study the focus was on the specific interaction between HUCs and spider silk. However, future research should determine the interaction between SMCs and spider silk as well as a combination of HUCs, SMCs and spider silk. A current drawback of spider silk for *in vivo* use is the difficulty of large-scale production. In addition, wetted spider silk fibers show supercontraction [59]. In this study, we used supporting weaving frames to overcome this problem. The demonstration that in an *in vivo* fascia replacement model supercontraction can be overcome by suturing the silk mesh into a defect before releasing it from the frame, encourages the *in vivo* use of spider silk without the presence of a supporting scaffold. [28]. Additionally, great efforts are made to overcome these issues with the intensive research on recombinant spider silk [34,60]. By tuning the structure to suite the physical and environmental demands of an application, mechanical properties could be adapted to the specific requirements. The next step would be to test this new biomaterial in pre-clinical models, in which, amongst others, effects of bladder environment on spider silk, such as degradation due to ureum and mechanical forces, cell and tissue formation and tensile forces affecting adherence and functionality, should be investigated. When such hurdles can successfully be taken, possibilities of spider silk as a reconstructive biomaterial for urinary tissues could be endless.
